# Human immunodeficiency virus is a driven factor of human papilloma virus among women: evidence from a cross-sectional analysis in Yaoundé, Cameroon

**DOI:** 10.1186/s12985-020-01340-y

**Published:** 2020-05-19

**Authors:** Samuel Martin Sosso, Michel Carlos Tommo Tchouaket, Joseph Fokam, Rachel Kamgaing Simo, Judith Torimiro, Aline Tiga, Elise Elong Lobe, Georgia Ambada, Achille Nange, Ezechiel Ngoufack Jagni Semengue, Alex Durand Nka, Valère Tala, Collins Chenwi, Aissatou Abba, Aude Christelle Ka’e, Bouba Yagai, Vittorio Colizzi, Alexis Ndjolo

**Affiliations:** 1 Chantal Biya International Reference Center for research on HIV/AIDS prevention and management (CIRCB), Yaoundé, Cameroon; 2 School of Health Sciences, Catholic University of Central Africa, Yaoundé, Cameroon; 3 University of Yaoundé I, Yaoundé, Cameroon; 4 University of Rome “Tor Vergata”, Rome, Italy; 5 Evangelical University of Bandjoun, Bandjoun, Cameroon

**Keywords:** HPV, HIV, Women, Yaoundé

## Abstract

**Background:**

Human papillomavirus (HPV) is the leading cause of cervical cancers, causing 270.000 deaths annually worldwide of which 85% occur in developing countries with an increasing risk associated to HIV infection. This study aimed at comparing HPV’s positivity and genotype distribution in women according to their HIV status and determinants.

**Methods:**

A comparative study was carried out in 2012 at the Chantal BIYA International Reference Centre (CIRCB) among 278 women enrolled consecutively at the General Hospital and the Gynaeco-Obstetric and Paediatric Hospital of the City of Yaoundé. HPV genotyping was performed by real-time PCR, HIV serological screening by serial algorithm, CD4 T cell phenotyping by flow cytometry and HIV viral load by Abbott m2000RT. Statistical analyses were performed using Microsoft Excel 2016 and Graph Pad version 6.0 software; with *P* < 0.05 considered statistically significant.

**Results:**

Globally, mean age was 37 ± 3 years; median CD4-count for HIV+ was 414 cells/mm^3^ [IQR: 264.75–588] and median viremia was 50 RNA copies/mL [IQR: < 40–8288]. Overall HPV rate was 38.49% (107/278); 58.88% for single women vs. others (28.97% married, 2.80% divorced, 9.34% for widows), OR: 2.164; *p* = 0.0319. Following HIV status, HPV rate was 43.48% (80/184) among HIV+ vs. 28.72% (27/94) among HIV- (OR: 1.937; *p* < 0.0142); HPV genotypes among HIV+ vs. HIV- were respectively distributed as follows: genotype 16 (3.75% vs. 0.00%, *p* = 0.57), genotype 18 (3.75% vs. 3.70%, *p* = 1.00), co-infection 16 and others (8.75% vs. 7.40%, *p* = 1.00), co-infection 18 and others (8.75% vs. 11.11%, *p* = 0.71), co-infection 16, 18 and others (2.50% vs. 0.00%, p = 1.00) and other genotypes (72.50% vs. 77.78%, *p* = 0.80). Among HIV+ participants, HPV rate following CD4 was 62.88% (61/97) for CD4 < 500 vs. 35.71% (20/56) for CD4 ≥ 500 (OR: 3.05; *p* = 0.0012) while HPV rate following HIV viremia was 42.71% (41/96) with < 1000 RNA copies/ml vs. 66.00% (33/50) with > 1000 RNA copies/ml (OR = 0.384; *p* = 0.009).

**Conclusion:**

In Yaoundé, HPV rate appear to be very high, with higher rates of genotypes other than 16 and 18. In the event of HIV infection, the risk of HPV positivity is two times higher, favoured essentially by immunodeficiency. Thus, HIV-infected women should be closely monitored to prevent the emergence of cervical cancer.

## Background

Human papilloma viruses (HPV) are non-enveloped DNA viruses that represent the most widespread sexually transmitted infection worldwide, with more than 200 species. HPV genotypes are responsible for 95% of the occurrence of cervical cancer in women [1]. Notably, HPV causes benign and malignant tumours in humans and viral genotypes that are oncogenic are classified as high-risk (HR-HPV) or low-risk oncogenic HPV (LR-HPV) [2]. Although all HR-HPV genotypes have oncogenic potential, some are more frequently involved in cancers than others. Thus, HPV 16 and 18 represent approximately 70% of all cervical cancers worldwide, followed by HPV 31, 33, 45, 52, 58 and 68 which are among the ten most commonly isolated HR-HPV genotypes from cervical tumor biopsies [3, 4].

Indeed, cervical cancer is known to be a viro-induced neoplasm and that HPV is the causal agent in 99% of cases [4]. According to the World Health Organization (WHO), cervical cancer will kill more than 443,000 people worldwide each year by 2030, and 98% of these deaths will occur in developing countries, particularly in sub-Saharan Africa (SSA) where the HIV epidemic and other risk factors further increase the burden of this cancer [5, 6]. In SSA, HPV and HIV co-infection is commonly found and contributes to a rapid disease progression toward cervical cancer [4]. Importantly, HIV-positive women, especially those who are severely immunocompromised, are 5 times more likely to develop the cervical cancer than their HIV-negative peers.

Even though little is known on HPV genotypes circulating among HIV-infected women in SSA countries, recommendations for HPV vaccination in the population of people living with HIV (PLHIV) are still unclear [7]. Of note, Generally 66% of infections are cleared at 12 months and this increases to 90% at 24 months [8]. For 10% of HPV-infected individuals, there is viral persistence that remains at latency. In this frame, immunodeficiency may be the cause of viral persistence and reactivation subsequently [7, 9].

Factors that contribute to HPV infections include: early sexual activity, multiple sexual partners, smoking, prolonged oral contraception and immuno-compromission (due to HIV essentially) [9]. HIV infection also promotes HPV infection at molecular and cellular levels during different phases of HPV replication cycle such as, penetration into the target cell, HPV multiplication and HPV immune evasion from the host’s defences. Direct consequences are clinical manifestations of HPV-associated dysplasia, which has been recognized in early HIV epidemics [10]. As reported by Kevin A. Ault in 2006, High-risk, oncogenic HPV types (including HPV 16 and HPV 18) are associated with 99.7% of all cervical cancers [11]. A recent study conducted in Chad during the year 2018 showed that 11 genotypes of HR-HPV were detected in nearly 70% of the study population, and HR-HPV 58 genotype was the most prevalent (22.9%) [12]. In Cameroon, out of 14,000 new cases of cancer reported in 2016, cervical cancer occupied the second leading position of cancers among women (23.2%) [12, 13]. These observations are very concerning and suggest a high burden of HPV in these settings where HIV is also highly prevalent [14, 15]. We therefore hypothesised that HPV-infection is significantly higher among Cameroonian HIV-seropositive women as compared to their seronegative peers. Thus, in the light of evidence generated from countries with similar programmatic challenges [14, 16], understanding the burden of HPV among women, its circulating genotypes and the effect of HIV-infection on HPV-positivity, might help in shaping HPV preventive measures according to local realities in Cameroon.

Our study objective was to compare the rate of HPV-positivity according to HIV-serostatus, and to determine other predictors of HPV-positivity.

## Methods

### Study design and setting

A cross-sectional study was carried-out in 2012 from women attending the General Hospital and the Gyneco-obstetrical Hospital, both located in Yaoundé, the capital city of Cameroon. For all enrolled women, blood and cervical samples were collected. HIV screening tests, CD4 lymphocyte counts, HIV viral load, and HPV genotyping were performed at the Chantal BIYA International Reference Centre for research on HIV/AIDS prevention and management (CIRCB) in Yaoundé, Cameroon (http://circb.cm/btc_circb/web/). Briefly, CIRCB is a reference centre for HIV/AIDS, performing laboratory analysis with external quality controls and proficiency testing for HIV screening (CDC DTS), HIV early infant diagnosis (CDC PT program), CD4 and viral load (QASI, Canada).

### Sampling strategy

Following a consecutive non-probabilistic sampling method, a minimum sample size of 272 was required to meet the study outcomes. Consenting women, sexually active, aged 18 years and above, were eligible for the study. However, pregnant women or those who had undergone a total hysterectomy were not considered for inclusion. After consenting, a standard questionnaire was administered to all participants, covering socio-demographic characteristics, gynaeco-obstetrical and reproductive history. Whole blood and cervical samples were collected; cervico-vaginal smear reading was done; HIV screening was performed following a serial algorithm as per the national guidelines, plasma HIV-1 viral load (solely for HIV+ cases) was done using the Abbott m2000 RT-PCR platform, enumeration of CD4 T lymphocyte was performed by using the FACS Calibur of Becton Dickinson.

### HPV genotyping

For HPV genotyping, the Abbott Real Time HR-HPV test was used. Briefly, viral DNA was extracted and amplified using the Abbott real-time polymerase chain reaction assay, with simultaneous detection and genotyping of HPV 16, HPV 18, and a pooled detection of 12 other HR-HPV genotypes (HPV 31, HPV 33, HPV 35, HPV 39, HPV 45, HPV 51, HPV 52, HPV 56, HPV 58, HPV 59, HPV 66 and HPV 68).

### Statistical analysis

Data were collected using Excel 2016, and analyses were performed using Epi-info version 7 and Graph pad prism version 6. Odd ratio (OR) was calculated to determine whether the variable was a risk or protective factor. The confidence interval (CI) for the statistical tests was set at 95%, and the null hypothesis rejected at a 5% threshold. Chi square or Fisher-Exact tests were used whenever appropriate. *P*-values ≤0.2, obtained in uni- or bi-variate analysis, were considered in the multivariate analysis.

### Ethics considerations

Ethical clearance was obtained from the CIRCB Ethics Committee (ref N^°^1810), and administrative authorization was provided by the facilities where the study was conducted. Written informed consent was provided by each participant, and results were delivered free of charge to participants for their clinical benefits.

## Results

### Sociodemographic and bioclinical characteristics of the study population

A total of 278 eligible women were enrolled as participants in this study, thus meeting the minimum sample size of 272. According to HIV sero-status, 184 women were HIV-seropositive and 94 were HIV-seronegative. The mean age was 37 years ±3 years, similar between HIV-seropositive women (36.7 years) as compared to HIV-seronegative women (37.3 years). The most represented age range was [30–39] years (44%). Single women were the most represented (58.88%), followed by married women (28.97%), widows (9.34%), and divorced (2.80%).

### Immune status of HIV+/HIV- patients

Women with a normal immunity (CD4 ≥ 500) were fewer (35.71%) as compared to those immunocompromised (CD4 < 500) representing 62.88% of the general population. The overall median [IQR] CD4 count was 399 cells/mm^3^. According to HIV-serostatus, median CD4 was lower (354 [231–530 cells] cells/mm^3^) among HIV-seropositive women as compared to their sero-negative peers (414 [IQ 262–918 cells/mm^3^]). The non-significant difference in CD4 distribution according to HIV status reflects the systematic HIV diagnosis performed to all women consenting for participation, most of whom were still asymptomatic.

### HPV positivity rate and circulating genotypes

Overall, HPV positivity rate was 38.49% (107/278), indicating a high burden of HPV in the study population (Table 1). According to HIV serostatus, HPV prevalence was significantly higher in the group of HIV-seropositive women (43.47% vs. 28.42%, *p* = 0.019), indicating that HIV-infection increasing by about 8 folds more risks of acquiring HPV as compared to those free of HIV (OR: 8.609, 95% CI: 2.841–27,338).

**Table 1:** HPV positivity according to HIV serostatus

HPV HIV	HPV + n (%)	HPV – n (%)	Total	***P***-value
**HIV+**	80 (43.47)	104 (56.53)	184	
**HIV-**	27 (28.42)	67 (70.53)	95	0.019
**Total**	107 (38.49)	171 (61.51)	278	

*HPV* human papillomavirus, *HIV* human immunodeficiency virus

### Diversity of HPV genotypes in infected women

**Fig. 1:**
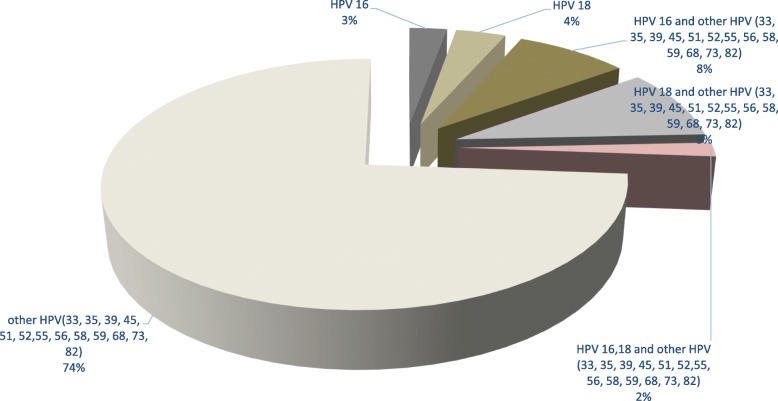
Distribution of HPV genotypes Legend, HPV: human papillomavirus; others refer to oncogenic HPV other than the 16 and 18 genotypes identified by the molecular detection assay

Several HPV genotypes were identified in the study population. Interestingly, 74% of women were infected by non-discriminated high-risk genotypes (31, 33, 35, 39, 45, 51, 52, 56, 56, 58 and 11 others) as per the genotyping assay. Surprisingly, only 3 and 4% of the study population were infected solely with HPV 16 and HPV 18 respectively (Fig. 1).

### Factors associated with HPV-infection

The age range 20–29 years was the most infected by HPV (51.66%). This latter prevalence was significantly higher when compared with that obtained in those aged 40–49 years (25.00%); *p* = 0.03. Overall, HPV positivity decreased as participants grow older (Table 2).

**Table 2:** HPV positivity by age

HPV Age (years)	HPV+ n (%)	HPV- n (%)	Total	***P***-value
**20–29**	31 (51.66)	29 (48.33)	60	Ref
**30–39**	49 (41.52)	69 (58.47)	118	0.20
**40–49**	16 (25.00)	48 (75.00)	64	0.03
**50–75**	11 (30.55)	25 (69.44)	36	0.64
**Total**	107 (38.49)	171 (61.51)	278	

*HPV* human papillomavirus, *Age* different age groups

According to marital status, the highest HPV positivity rate was recorded among divorced women (60%), and this latter prevalence was significantly higher when compared to those that were single (*p* = 0.04) (Table 3).

**Table 3:** HPV Association and Marital Status

HPV Marital status	HPV + n (%)	HPV – n (%)	Total	***P***-value
**Single**	63 (43.75)	81 (56.25)	144	Ref
**Married**	31 (30.69)	70 (69.31)	101	0.36
**divorced**	3 (60.00)	2 (40.00)	5	0.04
**Widow**	10 (35.71)	18 (64.29)	28	0.55
**Total**	107 (38.49)	171 (61.51)	278	0.32

*HPV* human papillomavirus, *Marital status* different marital status

Participants with poor immune status (CD4 ≤ 500) had a significantly higher rate of HPV positivity as compared to those with a normal immunity (62.88% versus 35.71% respectively, *p* = 0.046). Interestingly, the lower the CD4-count, the higher the rate of HPV positivity (Table 4).

**Table 4:** HPV-infection according to immune status

HPV CD4 (cells/mm^**3**^)	HPV + n (%)	HPV – n (%)	Total	***P***-value
**500–1600**	20 (35.71)	36 (64.29)	56	Ref
**350–499**	26 (55.31)	21 (44.68)	47	0.046
**200–350**	15 (57.69)	11 (42.31)	26	0.844
**< 200**	20 (83.33)	4 (16.67)	24	0.048
**Total**	81 (52.94)	72 (47.05)	153	

Overall, HPV positivy seemingly increased with increasing values of viral loads. 42.71% (41/96) with < 1000 RNA copies/ml vs. 66.00% (33/50) with > 1000 RNA copies/ml (OR = 0.384; *p* = 0.009). However, these increments were not statically significant (Table 5).

**Table 5:** HPV positivity rates by HIV viremia

HPV HIV viremia (Log copies/mL)	HPV+ n (%)	HPV- n (%)	Total	***P***-value
**Undetectable (< 1.60)**	25 (39.68)	38 (60.31)	63	Ref
**1.60–2.99**	25 (49.01)	26 (50.98)	51	0.34
**≥ 3.00**	38 (64.40)	21 (35.59)	59	0.12
**Total**	88 (50.88)	85 (49.13)	173	

*HPV* Human papillomavirus, *viremia* different variation of HIV viral load

After adjusting for CD4, viral load and HIV status, only HIV-infection was 12 times more likely to increase the risk of acquiring HPV-infection (*p* = 0.0001), as shown in (Table 6).

**Table 6:** Multi-variate analyses

Variable	Odds Ratio	Confidence interval 95%		***P***-value
**CD4**: **500–1600**	0.7323	0.2211	2.4260	0.6102
**CD4**: **350–499**	1.4735	0.4490	4.8361	0.5226
**VL Undetectable**	0.3839	0.1273	1.1580	0.0892
**VL 1.60–2.99**	0.5316	0.1109	2.5487	0.4295
**HIV status**	12.5000	4.0000	39.3000	< 0.0001

*VL* viral load of HIV

## Discussion

The purpose of our study was to compare the rate of HPV positivity among HIV-positive women with that of HIV-negative women, and to determine predictors of HPV-infection. Though all women participating in this study were living in the centre region of Cameroon, the population living in this region is very cosmoplite, as they come from all regions of the country. Therefore, evidence from this geographical setting could mitigate the national representativeness.

Out of a sample of 278 women enrolled, the mean age (37 years) mainly represents the most sexually active population and therefore having chronic exposure to sexually transmitted infections, including HPV. These results are similar to those obtained in Cameroon with Kabayene et al. (mean age of 38.5 years) and in Chad (mean age of 35 years) [12, 17, 18].

According to marital status, single women were the most represented (58%), a rate above the national proportion published in 2005 by the Central Bureau of Censuses and Population Studies in Cameroon where single women had a percentage of 41.46% [14]. the difference could be explained by changing overtime, as the social realities in the population could justify the increasing rate of single women [15].

The median CD4 count was lower in the frame of HIV-infection (354 versus 414 cells/mm^3^] in HIV-negative women. Median CD4 obtained in HIV-free population was surprising; however, this could be justified by the fact that these women attending the health facility (likely due to illed-health for some of them) [14, 15, 19]. Furthermore, it was previously reported that CD4 levels could vary with different conditions, including but not limited to stress, smoking, menstrual cycle, contraceptive pill, physical activity, etc. [20]

The HR-HPV positivity rate was 38.48%, with very low rate of genotypes 16 and 18. 3% for mono-infection with HPV genotype 16 and 4% for mono-infection with HPV genotype 18. This suggests that other genotypes are drivers of HPV-infection in the Cameroonian context and need to be depict among cervical cancer cases, especially in the frame of HIV-infection [14, 20]. Our findings are comparable to those found in Central Africa Republic [21], which suggest a predominance of non-vaccine HR-HPV different from those found in Western Europe and North America [22, 23]. Interestingly, in a systematic review conducted by Doh et al.*,* the prevalence of pure HPV 16 and 18 was 6.25 and 3.28% respectively, a finding very similar to our report showing evidence of 3% HPV 16 and 4% HPV 18 within the same country [24]. This thererfore stresses the fact that a bivalent vaccine would have very little predictive effectiveness within the population of Cameroonin women. Co-infection with HIV infection is a factor facilitating carcinogenesis associated with HR-HPV infections. Prospective studies have reported a higher incidence of HPV among HIV-positive women compared to HIV-negative women [1, 14, 16]. Co-infections of several HR-HPV genotypes was also been recorded, similar to previous reports [25]. Multi-infections are often associated with viral persistence, especially if co-present with HPV 16.

HIV is confirmed as a contributing factor to HPV-infection (*p* < 0.019), also confirmed by multi-variate analysis. As expected, women infected with HIV have a weaker immune status [26], which henceforth increases risk of acquiring HPV [27, 28]. This calls for the need to regularly test people with HPV infection for HIV [27].

Younger age are at higher risk of acquiring HPV-infection, likely due to sexual habits [29]. Regarding marital status, divorced women stand at high risk of acquiring HR-HPV. The lack of statiscal power limits the interpretation of this finding. Nonetheless, divorced women might be prone to multiple sexual partners, leading to HPV-infection [3, 30, 31]. The lack of synergistic effect between the HIV viral load and HPV-infection simply confirmed that HIV-infection interfers with HPV through immunodeficiency induced by HIV, and not via a direct viral-viral interaction [32, 33].

After adjusting the different variables based on a multi-variate analysis, HIV status remains the independent factor associated with the risk of infection with this high-risk oncogenic HPV (12 times higher risk), as previously demonstrated [34, 35]. Similar to our findings, the study by Tartaglia E. et al. also supports HIV-status as the main independent factor associated with risk of HPV infection among women, likely due to immunodeficiency. In other words, following multivariate analysis, none of the other parameters (except CD4 which is strongly related to the HIV condition) were found to be significantly associated with HPV infection [36].

Our study has some limitations. For instance, the low CD4-count in uninfected women was surpoirsing. This could simply be due to the consecutive sampling strategy that might lead to a certain degree of selection bias. Of note, the number of HIV-uninfected women was low; the presence of other comorbidities or infections such as hepatitis B, hepatitis C, and tuberculosis, could also affect the immune status. Unfortunately, diagnosis of these clinical conditions was not covered within the frame of the study. Further studies could therefore considered these aspects. Also, we have enrolled our patients only in the city of Yaounde and the reference molecular technique used for HPV detection made it possible to identify only genotypes 16 and 18. Given the high rate of other genotypes, it becomes essential to characterize this group. This therefore calls for sequencing for a better molecular characterization toward a rational selection of HPV vaccine candidate for the country.

## Conclusion

In this sub-population of Cameroonian women, HPV burden appears to be very high, with higher rates of genotypes other than the commonly known 16 and 18. Interestingly, in the event of HIV infection, the risk of HPV-infection becomes higher, favoured essentially by immunodeficiency. Further HPV gentopic studies, coupled to vaccine trials, would are needed to ensure effective prevention of cervical cancer among HIV-infected women.

## Data Availability

The dataset is available from the corresponding author.

## Abbreviations

AIDS: Acquired immunodeficiency syndrome
CD4: Cluster of differentiation 4
CIRCB: “Chantal BIYA” International Reference Centre for research on HIV/AIDS prevention and management
DNA: Deoxyribonucleic acid
HIV: Human Immunodeficiency Virus;
HPV: Human papilloma virus
HR-HPV: High risk oncogenic Human Immunodeficiency Virus
LR-HPV: Low risk non-Human Immunodeficiency Virus
PLHIV: People living with HIV
RT-PCR: Real-time polymerase chain reaction
WHO: World health organization
